# Early warnings for multi-stage transitions in dynamics on networks

**DOI:** 10.1098/rsif.2022.0743

**Published:** 2023-03-15

**Authors:** Neil G. MacLaren, Prosenjit Kundu, Naoki Masuda

**Affiliations:** ^1^ Department of Mathematics, State University of New York at Buffalo, Buffalo, NY 14260-2900, USA; ^2^ Computational and Data-Enabled Science and Engineering Program, State University of New York at Buffalo, Buffalo, NY 14260-5030, USA

**Keywords:** complex networks, early warning signals, critical transitions, tipping points, dynamics on networks

## Abstract

Successfully anticipating sudden major changes in complex systems is a practical concern. Such complex systems often form a heterogeneous network, which may show multi-stage transitions in which some nodes experience a regime shift earlier than others as an environment gradually changes. Here we investigate early warning signals for networked systems undergoing a multi-stage transition. We found that knowledge of both the ongoing multi-stage transition and network structure enables us to calculate effective early warning signals for multi-stage transitions. Furthermore, we found that small subsets of nodes could anticipate transitions as well as or even better than using all the nodes. Even if we fix the network and dynamical system, no single best subset of nodes provides good early warning signals, and a good choice of sentinel nodes depends on the tipping direction and the current stage of the dynamics within a multi-stage transition, which we systematically characterize.

## 1. Introduction

A characterization of complex systems is dependence among components, which often leads to surprising, nonlinear behaviour. One important nonlinear phenomenon is that of a tipping point: a transition in which stable aspects of the system suddenly shift to a drastically altered state when the system’s environment changes by a small amount; recovery from the altered state is typically difficult. Tipping points have been described in, for example, the switch from clear to turbid water in lake ecosystems [1], changes in fish community composition [2], alterations in global climate regimes [3] and in the progression of disease [4,5]. This shared feature of such disparate systems can be described mathematically by bifurcations, and several early warning signals—statistical indications that a bifurcation point is nearby—have been developed that attempt to anticipate such transitions. These early warning signals rely on a process called critical slowing down: systems recover from perturbations more slowly near a bifurcation point [6]. Critical slowing down results in predictable signatures in time-series data, including increasing variance and autocorrelation, and it is these signatures that are used to construct early warning signals. Early warning signals based on the critical slowing down phenomenon have been validated in several model systems [2,7], and their practical utility has been demonstrated in e.g. predicting electrical grid failures [8] and reversing cyanobacterial blooms [9].

Many systems showing tipping points can be modelled by a network in which a node represents a dynamical system and different dynamical systems interact through the edges of the network [10]. Studying tipping points in such systems is an integral part of studying network robustness and resiliency [11]. An example with applications in conservation ecology is the anticipation of a breakdown in mutualistic species networks [12–14]. In such models, species populations are typically represented by stochastic differential equations interacting through a bipartite network of plants and pollinators [15,16] or a unipartite projection focusing on only plants or pollinators [12,17]. Early warning signals can then predict major adjustments in species composition [12] or population collapse [14]. Similarly, exploiting information on interactions between weather patterns in different regions may improve the forecasting of climate tipping points [18].

In fact, the inherent heterogeneity in networked systems may make tipping points more complex. Specifically, multi-stage transitions, in which not all components transition to an alternative state at the same parameter values, may be the rule rather than the exception in networks with certain features [19,20]. Multi-stage transitions have been documented in studies of mutualistic species dynamics [12,14] and climate systems [18], and are consistent with evidence from human commensal bacteria [21] and social upheaval [22]. The ability to anticipate multi-stage transitions would thus have applications in many fields.

A variety of methods have been proposed to provide early warning of tipping points on networks. Examples include aggregations of univariate (i.e. single-node) early warning signals and explicitly multivariate methods such as measures derived from a principal component analysis (PCA) of state variables [11,23]. However, most of the available early warning signals for networks treat the network as a united entity and do not exploit the fact that a network is composed of subsystems that may show different dynamics and provide different early warning signals. There are some notable exceptions. First, Chen *et al.* [24] used cross-correlations to identify clusters of nodes that were more sensitive to an approaching bifurcation than the network as a whole. Although Chen *et al.* exploited network heterogeneity for constructing early warning signals, they did not consider multi-stage transitions. Second, Lever *et al.* developed PCA methods to predict the direction and magnitude of change for each node’s state after a bifurcation [12]. Lever *et al.* noted parameter ranges for their model in which multi-stage transitions were possible and that the early warning signal they proposed tended to correctly anticipate the first transition. However, Lever *et al.* noted that their method was less reliable for describing further nodes’ transitions—the multi-stage transition. Third, Aparicio *et al.* [14] used network control theory—rather than system dynamics—to identify nodes that would be capable of providing a reliable early warning signal. They also identified parameter values that caused multi-stage transitions in their model and also found that their method underperformed in those regions. In contrast to Lever *et al.*’s method, Aparicio *et al.*’s method tended to miss early transitions of nodes but correctly predicted the final collapse. Based on the ubiquitousness of multi-stage transitions in networks, discussed above, there is a need for early warning signals that can provide alerts for each of the major tipping points within a multi-stage transition that a networked system may experience.

In the present study, we build on key points from these three studies—namely that (i) some nodes may be more informative about impending transitions than others and (ii) information may be available in the network structure or dynamics with which to anticipate multi-stage transitions—to investigate early warning signals for multi-stage transitions in tipping dynamics on networks. We find that traditional early warning signals are in fact able to provide early warning in a network undergoing a multi-stage transition. Using knowledge of the network allows us to choose ‘sentinel’ nodes, i.e. node sets that can provide early warning more efficiently than using all nodes in terms of the number of nodes we must observe. Furthermore, it is often the case that such early warning signals even improve in accuracy.

## 2. Methods

### 2.1. Model

Consider an undirected and unweighted network of *N* nodes and denote its adjacency matrix by *A* = (*a*_*ij*_) with *a*_*ii*_ = 0 and $a_{ij}=a_{\;ji}\in \{0,1\}\;\forall \;i,j\in \{1,\ldots ,N\}$. We simulate the stochastic dynamics of a coupled double-well model on networks given by

2.1 $$\frac{dx_{i}}{dt}=-(x_{i}-r_{1})(x_{i}-r_{2})(x_{i}-r_{3})+D\sum_{\;j=1}^{N}a_{ij}x_{j}+s\xi_{i},$$

where *x*_*i*_ is the state of node *i*; *r*_1_, *r*_2_ and *r*_3_ are parameters that control the location of the equilibria and satisfy *r*_1_ < *r*_2_ < *r*_3_; *D* (≥0) is the coupling strength; and *sξ*_*i*_ is a Gaussian noise process with standard deviation *s*. The first term is the derivative of a fourth-order polynomial representing a double-well potential. In the uncoupled and noiseless case, it produces lower and upper stable equilibria at *x*_*i*_ = *r*_1_ and *x*_*i*_ = *r*_3_, respectively, and an unstable equilibrium at *x*_*i*_ = *r*_2_, and it also creates hysteresis. Unless we state otherwise, we set (*r*_1_, *r*_2_, *r*_3_) = (1, 4, 7). The coupling term $D\sum_{\;j=1}^{N}a_{ij}x_{j}$ shifts *x*_*i*_ at the stable equilibria out of *x*_*i*_ = *r*_1_ = 1 or *x*_*i*_ = *r*_3_ = 7. In addition, the noise term *sξ*_*i*_ lets *x*_*i*_ jitter around the stable equilibria obtained in the absence of noise. We, therefore, consider that nodes with *x*_*i*_ < 2.268 are in the lower state and *x*_*i*_ > 2.268 are in the upper state. We selected this threshold value for *x*_*i*_ because the cubic term in equation (2.1) has an inflection point at *x*_*i*_ ≈ 2.268 in the absence of the coupling term, demarcating a basin of attraction for the lower stable point at *x*_*i*_ = 1. We numerically verified that we can reliably classify *x*_*i*_ into the lower and upper stable equilibria with these threshold values even in the presence of the coupling term (see electronic supplementary material, figure S1). Equation (2.1) represents dynamics of species abundance [12] or climates in interconnected regions [18]. We primarily consider *D* as a bifurcation parameter. A possible mechanism underlying variation in *D* is the volume of moisture moving from one climate basin to another [18].

For applications such as species loss in population ecology, one is interested in beginning with the upper state, which corresponds to the situation in which all the species are abundant, and gradually varying a parameter value to anticipate transitions of various nodes to their lower states [6]. For example, a transition to the lower state could correspond to the collapse of a species’ population. To validate the relevance of multi-stage transitions and early warning signals in this scenario, we consider an extension of equation (2.1) given by

2.2 $$\frac{dx_{i}}{dt}=-(x_{i}-r_{1})(x_{i}-r_{2})(x_{i}-r_{3})+D\sum_{\;j=1}^{N}a_{ij}x_{j}+u+s\xi_{i}.$$

Variable *u* is a stressor that directly and uniformly influences all nodes. An increase in *u* represents, for example, increased global mean temperature [18] or degradation of the local environment causing increased mortality for all species [12]. With equation (2.2), we hold either *D* or *u* constant and vary the other as the bifurcation parameter.

### 2.2. Numerical simulations

Unless we state otherwise, we used *D* as the bifurcation parameter and began simulations with all nodes in the lower state. For the given network and the value of *D*, we started the dynamics from the initial condition *x*_1_ = · · · = *x*_*N*_ = 1. For any given value of *D*, we integrated equation (2.1) using the Euler–Maruyama method with time step Δ*t* = 0.01 for 50 time units (TU) to allow {*x*_1_, …, *x*_*N*_} to relax to an equilibrium. In fact, allowing 50 TU was sufficient except in rare cases in which some nodes changed their macroscopic state (i.e. lower versus upper state) after 50 TU due to dynamical noise. We then continued simulating the dynamics for a further 25 TU to take samples from {*x*_1_(*t*), …, *x*_*N*_(*t*)} for calculating early warning signals. We used *s* = 0.05 except where noted.

To determine whether or not early warning signals increase prior to transitions of various nodes from their lower state to upper state, we conducted sequences of the above simulations for a given network and set of parameters. Each sequence began with *D* = 0.01. After we simulated the dynamics for 75 TU in total and calculated early warning signals, we increased *D* by 0.005, reset $x_{i}\;\forall \;i$ to the initial condition, ran the simulation with the new value of *D*, and calculated early warning signals from the new *x*_*i*_(*t*). We continued this procedure (i.e. increasing *D* by 0.005 and running a new simulation) until at least 90% of nodes reached the upper state at equilibrium.

In simulations with *D* as the bifurcation parameter but with the nodes beginning in the upper state, we set $x_{i}=7\;\forall \;i$ and *u* = −15. In this case, we consider that nodes with *x*_*i*_ < 5.732 are in the lower state and *x*_*i*_ > 5.732 are in the upper state; note that equation (2.1) has a second inflection point at *x*_*i*_ ≈ 5.732 in the absence of the coupling term. We initially set *D* = 1 and decreased *D* by 0.005 in each simulation, continuing until greater than 90% of nodes transitioned to the lower state at equilibrium. All other parameters were the same regardless of whether we began simulations with the nodes at the upper or lower state.

This simulation method attempts to ensure that we always study the system at equilibrium and has been used in previous studies of tipping points on networks (e.g. [18]). De-trending or other preprocessing of data from the simulations is, therefore, not needed: by the time we take data from each simulation, the system is stationary by design (cf. [25] for a different simulation method, for which de-trending is required).

### 2.3. Early warning signals

At each value of *D*, we calculated the following early warning signals [23,25] from *M* = 250 equally spaced samples of {*x*_1_(*t*), …, *x*_*N*_(*t*)} with *t* ∈ (50, 75], i.e. with *t* ∈ {50.1, 50.2, …, 75.0}:

— The dominant eigenvalue *λ*_max_ of the covariance matrix, of which the (*i*, *j*) entry is the covariance of {*x*_*i*_(50.1), *x*_*i*_(50.2), …, *x*_*i*_(75)} and {*x*_*j*_(50.1), *x*_*j*_(50.2), …, *x*_*j*_(75)}.
— The standard deviation of each *x*_*i*_(*t*) estimated from the *M* samples.
— The lag-1 autocorrelation of each *x*_*i*_(*t*), defined as $(\sum_{m=1}^{M-1}$ $\left(x_{i,m}-{\bar{x}}_{i})(x_{i,m+1}-{\bar{x}}_{i}\right))/(\sum_{m=1}^{M}{\left(x_{m}-{\bar{x}}_{i}\right)}^{2})$, where *x*_*i*,*m*_ ≡ *x*_*i*_ (50 + 0.1 *m*) and ${\bar{x}}_{i}=\sum_{m=1}^{M}x_{i,m}/M$.

To define an early warning signal for a given node set, we used both the maximum and the mean of the standard deviation and lag-1 autocorrelation in addition to *λ*_max_ calculated from the node set of interest. Therefore, we examine five different early warning signals for a given set of nodes (see §2.4 for the node sets).

We quantify the extent to which an early warning signal anticipates a bifurcation with the Kendall rank correlation, *τ*, between *D* before the bifurcation occurs and the early warning signal [26]. The reasoning behind using Kendall’s *τ* as a performance metric is as follows. Consider a range of *D* in which no nodes change state at equilibrium except at the final value of *D*. We refer to a range of *D* in which the number of nodes in the lower/upper state is constant as a stable range. Given our simulation protocol, *D* is linearly increasing in a stable range. If an early warning signal tends to increase as *D* increases towards the bifurcation point, indicating critical slowing down, then the early warning signal is considered to be useful in anticipating the bifurcation, and *τ* tends to be large. However, in the network dynamics that we are considering, there are potentially many values of *D* at which some nodes switch from the lower to the upper state. Therefore, we correlate *D* with a given early warning signal to obtain *τ* only within stable ranges of *D* having at least 15 unique values of *D*. We report the *τ* value averaged over all such stable ranges. For example, if there is no node transitioning from its lower state to the upper state for *D* ∈ {0.01, 0.015, …, 0.5}, *D* ∈ {0.505, 0.51, 0.515} and *D* ∈ {0.52, 0.525, …, 1}, some nodes transit from the lower to the upper state at *D* = 0.505, 0.52 and 1.005, and the transition at *D* = 1.005 makes the fraction of the nodes in the upper state exceed 0.9, then we calculated *τ* for the first and third ranges of *D* and took the average of the two *τ* values.

### 2.4. Node sets

We defined the following nine node sets for calculating the early warning signals:

— ‘All’ refers to the set of all nodes.
— ‘Lower State’ refers to the set of all nodes in the lower state at t=50 TU.
— ‘Upper State’ refers to the set of all nodes in the upper state at t=50 TU. If there are no nodes in the upper state, this node set is empty and early warning signals for this node set are undefined.
— ‘High Input’ refers to the *n* nodes that are largest in terms of $R_{i}=\sum_{\;j=1}^{N}a_{ij}{\bar{x}}_{j}$, where *i* is the index of an available node in the sense that it is still in its original macro state. For example, a lower-state node is an available node if nodes are initially in the lower state in a simulation. Note that such a node is available to transition to the upper state as *D* increases. We remind that ${\bar{x}}_{j}$ is the mean of *x*_*j*_ calculated over the *M* samples. We define the High Input node set based on the idea that a lower-state node with many neighbours or with neighbours in the upper state is more likely to transition from the lower to the upper state earlier than other nodes.
— ‘Low Input’ refers to the *n* nodes that are the smallest in terms of *R*_*i*_. As for High Input, we require that the *i*th node is in its original macro state. The Low Input node set reflects the observation that, if the nodes are initially in the upper state, then the node with the smallest contribution from the coupling term, i.e. those with smallest *R*_*i*_, would be the first to transition to the lower state as *D* decreases.
— ‘Lower Half’ refers to the set of lower-state nodes below the median in terms of *R*_*i*_; we do not use this node set when all the nodes are initially in the upper state in the simulation. If the nodes begin in the lower state, Lower Half nodes are the farthest from a bifurcation as one gradually increases *D*.
— ‘Random’ refers to the set of *n* nodes selected uniformly at random.
— ‘Large Correlation’ nodes are the top *n* nodes in terms of $R_{i}^{\prime }=\sum_{\;j=1;j\neq i}^{N}\mathrm{cor}(x_{i},x_{j}){\bar{x}}_{j}$, where the *i*th node is a lower-state node, and cor(*x*_*i*_, *x*_*j*_) is the Pearson correlation coefficient between *x*_*i*_ and *x*_*j*_ calculated over the *M* samples. This is an alternative for High Input when we do not have access to the network structure, i.e. the adjacency matrix.
— ‘Large Standard Deviation (Large s.d.)’ nodes are the *n* nodes with the largest standard deviation of *x*_*i*_ over the *M* samples. A node tends to have a larger standard deviation when it receives a larger input from the coupling term. Thus, the Large s.d. node set is also an alternative for High Input when we do not have information about the network structure.

The All node set corresponds to established early warning signal methods and is the most costly in terms of sampling effort. The High Input, Low Input, Random, Large Correlation and Large s.d. node sets require a limited number of nodes, which we set *n* = 5, and are, therefore, the least costly. The other node sets are variable in terms of the number of nodes. However, with the exception of the first stable range, the number of nodes used is typically much larger than *n* and much smaller than *N* across a wide range of *D*. All, Lower State, Upper State, Random, Large Correlation and Large s.d. do not use the information on the network structure, whereas High Input, Low Input and Lower Half do. Random, Large Correlation and Large s.d. are most economic in the sense that it only uses *n* nodes and does not require the network structure. We updated node set membership each time we change the value of *D*.

### 2.5. Networks

We conducted simulations on six model networks and 17 empirical networks; see the electronic supplementary material for details of the networks. We chose networks having the order of 100 nodes, similar in size to many empirical networks and small enough to be computationally feasible for our simulations. We chose model networks with a range of degree heterogeneities and with and without a planted community structure, including networks that show a multi-stage transition to different extents [20]. Empirical networks may have a variety of features difficult to capture with model networks and thus present hidden challenges to our methods. An example of our empirical networks is a dolphin social network [27]. In this network, the nodes are individual dolphins and two nodes are adjacent if individuals *i* and *j* were observed together more often than expected by chance. On such a network, *x*_*i*_ represents, for example, a behavioural state or possession of particular information.

### 2.6. Robustness analysis

We tested several variations of our methods to examine robustness under different scenarios. First, to test the robustness of these results with respect to the network structure, we conducted simulations on the 23 networks explained in §2.5. Ten of the 23 networks had at least two stable ranges, showing clear multi-stage transitions. We selected these 10 networks for further analysis.

Consider an early warning signal. On each of the 10 selected networks, we calculated *τ* between the early warning signal and *D* for each stable range of *D*. We then averaged *τ* over the stable ranges of *D*. We calculated such an averaged *τ* value 50 times, restarting simulations with a new random seed each time, for each of the three node sets (i.e. All, Lower State and High Input) and each network. Finally, we estimated a linear mixed effects model to predict the averaged *τ* value based on three levels of a node-set fixed effect variable (i.e. All as the reference, Lower State and High Input) with a random effect for network. We estimated the linear mixed effects model in this manner for each of the five early warning signals.

Second, we varied several simulation parameters on two arbitrarily selected networks. The adjusted parameters were the noise intensity (*s* ∈ {0.01, 0.1, 0.5}), the number of samples taken from each *x*_*i*_(*t*) when calculating early warning signals (*M* ∈ {25, 50, 150}), the double-well model parameters ($\left(r_{1},r_{2},r_{3})\in \{(1,3,5),(1,2.5,7),(1,5.5,7)\right\}$), and the duration *T* of the simulation before we start to sample {*x*_1_(*t*), …, *x*_*N*_(*t*)} to calculate the early warning signals at each value of *D* (*T* ∈ {25, 75, 100}).

Third, we altered the model itself, examining transitions from the upper to the lower state using equation (2.2).

### 2.7. Software

We conducted all simulations and analyses in R (v. 4.2); dependencies include the ‘igraph’ package (v. 1.3) for network analysis [28], the ‘nlme’ package (v. 3.1) for mixed effects statistical models [29] and the ‘parallel’ package (v. 4.2) [30] for parallel processing. Empirical networks were drawn from the ‘networkdata’ package [31]. Code and data to reproduce these analyses are available at https://github.com/ngmaclaren/doublewells.

## 3. Results

### 3.1. Multi-stage transitions and performance of early warning signals based on different node sets

**Figure 1.**
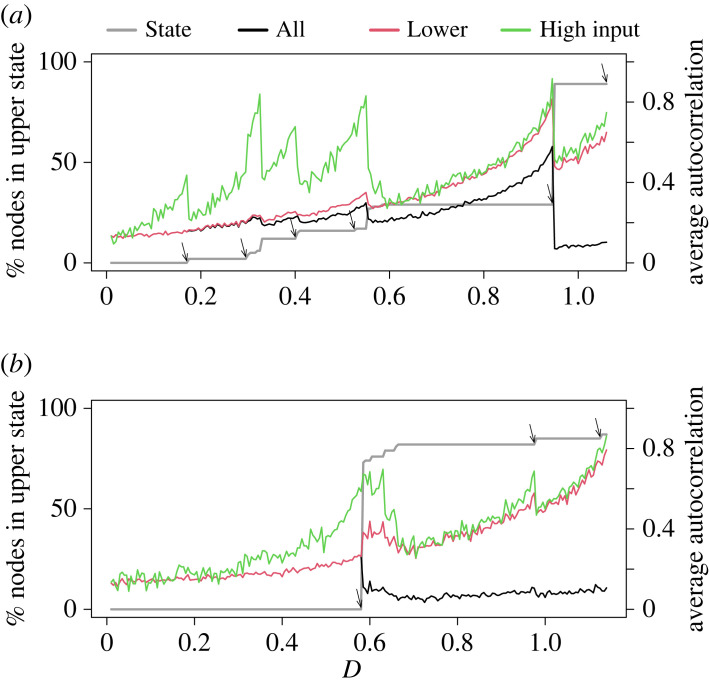
Multi-stage transitions when the nodes are initially in the lower state. We show the number of nodes in the upper state at equilibrium (grey), the average lag-1 autocorrelation of *x*_*i*,*t*_ calculated for all nodes (black), the nodes in the lower state (red) and the low-input nodes (green). The arrows mark transitions of some nodes at the ends of stable ranges. (*a*) A network with 100 nodes and a power-law degree distribution and (*b*) dolphin social network.

Let us first consider a network with 100 nodes and a power-law degree distribution generated by a configuration model, which we call the power-law network. We show by the grey line in figure 1*a* the proportion of nodes in the lower state in the equilibrium as a function of the coupling strength between nodes, *D*. The figure shows that more nodes tend to be in the upper state in the equilibrium when *D* is larger. Additionally, there are ranges of *D* in which relatively large changes in *D* do not induce transition of any node from the lower to the upper state at equilibrium. In other ranges of *D*, small changes in *D* trigger transitions of some nodes between macro states. In this manner, the noisy double-well model on this network shows a multi-stage transition. We also find a multi-stage transition when we use equation (2.2) and vary *u* instead of *D* as the bifurcation parameter (electronic supplementary material, figure S2).

Early warning signals appear to be sensitive to changes in *D*. Figure 1*a* also shows a typical early warning signal, i.e. the lag-1 autocorrelation of *x*_*i*_(*t*), averaged over three different node sets. The first, ‘All’ (black), corresponds to traditional early warning signals and refers to the set of all nodes. Within the stable ranges of *D*, the early warning signal value tends to increase as *D* increases. However, different nodes may be differently informative as to an impending transition. Both observed dynamics [12,24] and knowledge of network structure [14] may improve the accuracy of early warning signals or their efficiency in terms of the amount of observed signals necessary for the calculation. In fact, it may be more efficient to monitor nodes that are most likely to transition to an alternative state with a perturbation of a control parameter.

To show that monitoring sentinel node sets can be effective, figure 1*a* also displays the early warning signal calculated for the set of nodes in the lower state at t=50 TU (‘Lower State’, red) and the set of five nodes most likely to transition from the lower to upper state (‘High Input’, green). These latter nodes have many neighbours, are connected to nodes that have transitioned to the upper state, or both; they have the highest value of $R_{i}\equiv \sum_{\;j=1}^{N}a_{ij}{\bar{x}}_{j}$ by definition. Figure 1*a* shows that the sensitivity of the average autocorrelation to the increase in *D* towards the end of a stable range varies depending on the node set and the value of *D*. For example, there is a major sudden increase in the number of nodes in the upper state at equilibrium at *D* = 0.95. This transition is associated with, looking from left to right, a marked increase in the average autocorrelation of the nodes in each of the node sets at *D* just below 0.95 and a decrease in the average autocorrelation at *D* = 0.95. A similar tendency is present around the transitions of smaller batches of nodes at, for example, *D* = 0.175, 0.33 and 0.55. Changes in the average autocorrelation of the High Input nodes tend to be larger in absolute value than for the Lower State and All node sets, particularly at smaller values of *D*, but the overall range is similar in this network.

Figure 1*b* shows that the double-well model on a dolphin social network [27] also exhibits a multi-stage transition. See electronic supplementary material, figure S2 for similar results when *u* is the bifurcation parameter. Compared with the case of the power-law network, the dolphin network allows larger stable ranges of *D*, and the ranges of *D* in which small changes in *D* induce a transition of a notable fraction of nodes from the lower to the upper state are narrower. Similar to figure 1*a*, the autocorrelation tends to reliably increase in each stable range of *D* as we increase *D* towards the value at which some nodes transit from the lower to the upper state. In addition, the average autocorrelation based on the Lower State and High Input node sets apparently better signals such transitions than that based on all nodes, in the sense that the average autocorrelation increases more drastically as *D* increases towards the bifurcation.

**Figure 2.**
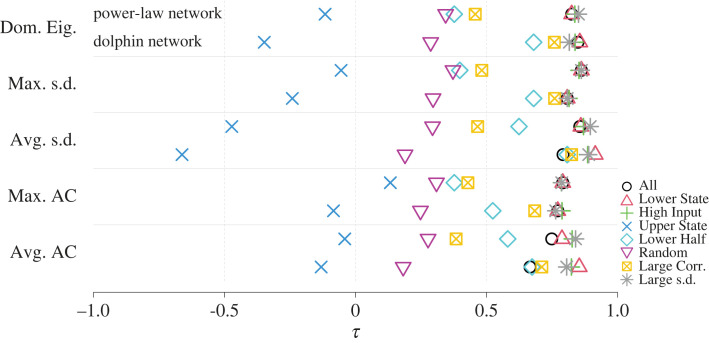
Kendall correlations (*τ*) between each of the five early warning signals and the coupling strength, *D*, for different sets of nodes. See main text for details of node set membership. Dom. Eig, dominant eigenvalue of the covariance matrix of all nodes in the node set; Max. s.d. and Avg. s.d., maximum and average standard deviation of *x*_*i*_; Max. AC and Avg AC, maximum and average autocorrelation of *x*_*i*_; Large Corr., the Large Correlation node set.

To quantify the performance of the average autocorrelation and other early warning signals, we computed the Kendall’s *τ* for each of the two networks used in figure 1 and for each of the five early warning signals calculated for each node set. We show the results in figure 2, which indicates that *τ* is high (i.e. greater than 0.65) across both networks and all five early warning signals and for All (circles), Lower State (triangles) and High Input (pluses) node sets. The *τ* values for each early warning signal in both networks are similar between Lower State and High Input, and they are higher than for All in a majority of cases. In addition to having a high average *τ* value, the High Input node set has *τ* > 0.7 for each major transition in both networks (see electronic supplementary material, section S4 and figure S6 for details). If we calculate the average autocorrelation for the nodes that actually changed state at each major transition, we of course find that the *τ* value for this retroactively identified node set is high. However, the High Input node set has almost the same performance, in terms of *τ* at each transition, as the nodes that actually changed state (electronic supplementary material, figure S6). Furthermore, by definition, early warning signals calculated with the Lower State and High Input node sets are more cost-efficient than those calculated with all nodes because the former use only a fraction of nodes. However, our typical simulations use samples of *x*_*i*_ at all *M* time points for both assigning nodes to node sets and calculating early warning signals. We performed additional simulations, described in electronic supplementary material, section S5, which only used the samples at the first 10 time points to determine node set membership. We then monitored the node set members for the full *M* samples including the first 10 samples for calculating early warning signals. Our results are robust to this decision, as we show in electronic supplementary material, figure S7. Finally, the High Input node set performs well even when we consider all node transitions, not just those occurring after a stable range (electronic supplementary material, figure S8).

Although the Lower State node set is both more accurate and efficient than the set of all nodes, this result does not imply that any nodes in the lower state provide a good early warning signal. To show this, we investigated early warning signals constructed from half of the lower-state nodes whose *R*_*i*_ score is the lowest—those with relatively few neighbours or few neighbours in the upper state. This node set, termed ‘Lower Half’ and shown by the diamonds in figure 2, typically yielded lower *τ* values than the All, Lower State and High Input node sets. This result implies that one needs to assemble an early warning signal from carefully chosen lower-state nodes such as those with large *R*_*i*_ values. Finally, Upper State (shown by the crosses in figure 2) and Random (shown by the inverted triangles) node sets are either negatively correlated or not correlated with *D*, reinforcing our claim that the choice of nodes to be observed is essential. In sum, our simulation results suggest that, with a proper choice of observed node set—including the case of observing all nodes—standard multivariate and aggregated univariate indicators reliably increased in value prior to several transitions of nodes from the lower to upper state, performing well throughout a multi-stage transition.

### 3.2. Robustness against variation in networks and parameter values

**Figure 3.**
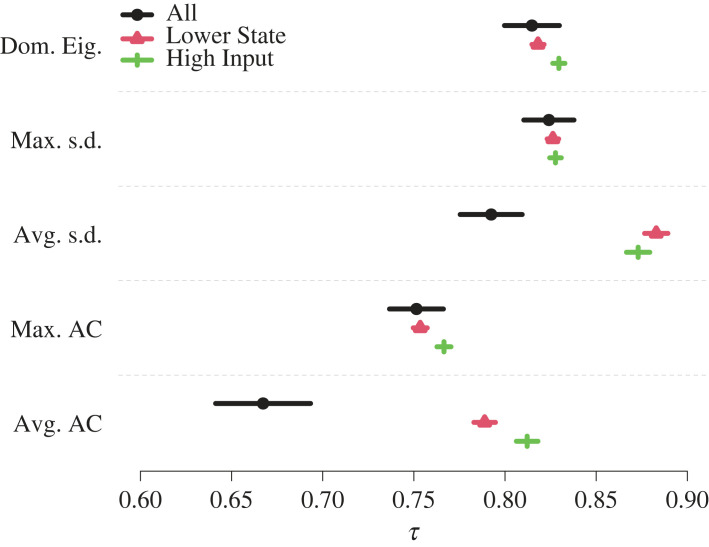
Predicted Kendall correlations (*τ*) for five early warning signals and three node sets, estimated by a linear mixed effects model with a fixed effect for node set and a random effect for network. The results are based on the 10 networks that have multiple stable ranges of *D* in our numerical simulations. Markers (All: circles, Lower State: triangles, High Input: pluses) signify the predicted *τ* value. The horizontal lines represent the 95% confidence intervals.

To quantitatively examine the dependence of *τ* on network structure, we constructed a linear mixed effects model explaining *τ* with a fixed effect of node set and a random effect of network (figure 3; see electronic supplementary material, section S9 for the statistical results). We found that the predicted *τ* is large (i.e. approximately larger than 0.75) across most networks, early warning signals and node sets; the combination of the All node set and the average autocorrelation early warning signal yielded a somewhat lower predicted *τ* value (i.e. 0.667). Variance-based methods (i.e. dominant eigenvalue and the maximum and average node-level standard deviation) tended to produce higher predicted *τ*, ranging between 0.792 and 0.828. The autocorrelation methods produced lower predicted *τ*, ranging between 0.667 and 0.766, although these values were still relatively high compared with other published results (e.g. [25,26]). The early warning signals based on the Lower State nodes were either no different (dominant eigenvalue, *p* = 0.050; maximum standard deviation, *p* = 0.173; and maximum autocorrelation, *p* = 0.290; uncorrected for multiple comparison) or better (average standard deviation, *p* < 10^−4^ and average autocorrelation, *p* < 10^−4^) than those based on all nodes. The early warning signals based on the High Input nodes improved over those based on all nodes (*p* < 10^−4^ for all the early warning signals except the maximum standard deviation, for which *p* = 0.025) on average but were not as good as those based on the Lower State nodes in the case of the average standard deviation (High Input: *τ* = 0.873, Lower State: *τ* = 0.883). The *τ* values at most moderately depended on the network structure. Specifically, the distribution of random intercepts for network had the smallest standard deviation in the estimated linear mixed effects models for the maximum standard deviation early warning signal (0.022, 2.7% of the magnitude of the intercept) and the largest standard deviation for the average autocorrelation early warning signal (0.041, 6.2%). These results are consistent with and generalize in terms of the variety of networks those shown in figure 2.

We then investigated the robustness of the results shown in figure 2 against changes in parameter values. The full results are shown in the electronic supplementary material, section S10. Consistent with previous results (e.g. [32]), decreasing the number of samples for calculating the early warning signal, *M*, has the strongest negative effect on the performance of early warning signals. We have also found that the average standard autocorrelation calculated from all nodes tends to perform worse than that calculated from the other node sets when the double-well equilibrium points are relatively close together (i.e. (*r*_1_, *r*_2_, *r*_3_) = (1, 3, 5) as opposed to (1, 4, 7)) or *r*_1_, *r*_2_ and *r*_3_ are not evenly spaced (i.e. (*r*_1_, *r*_2_, *r*_3_) = (1, 2.5, 7) or (1, 5.5, 7) as opposed to (1, 4, 7)). As expected, allowing more than 50 TU for the model to relax to an equilibrium does not markedly improve the performance of the early warning signals. Thus, with the notable exception of the effect of *M*, the performance of each early warning signal is in general fairly similar across the different parameter settings.

### 3.3. When we do not know the network structure

When we do not know the network structure, we cannot calculate *R*_*i*_, which uses the adjacency matrix, to identify High Input nodes. Therefore, we explored the use of a correlation-based index, *R*′_*i*_ (see §2.4 for the definition), to choose alternative sentinel nodes, called the Large Correlation nodes, and computed the same set of early warning signals. We show the results for the Large Correlation node set by the box-times symbols in figure 2. The Large Correlation node set performed worse than the High Input node set. This result is expected because High Input uses the information about the network structure, whereas Large Correlation does not. However, the Large Correlation node set performed better than the Lower Half and Random node sets. In fact, *τ* with the Large Correlation node set is reasonably large in the dolphin network, roughly ranging between 0.6 and 0.8, whereas it is low in the power-law network (i.e. *τ* < 0.5). The discrepancy between the results for the two networks is associated with the different fidelity with which the Pearson correlation matrix, cor(*x*_*i*_, *x*_*j*_), reflects the actual adjacency matrix (see electronic supplementary material, figure S9).

We also considered the nodes with the largest standard deviation in *x*_*i*_, called Large s.d., as another node set that does not need the information about the network structure. The rationale behind Large s.d. is that, when the *i*th node receives large input from other nodes, i.e. when *R*_*i*_ is large, the standard deviation of *R*_*i*_ should also be large because each *x*_*j*_ in equation (2.1) is fluctuating due to dynamical noise. A large fluctuation in *R*_*i*_ is expected to make the standard deviation of *x*_*i*_ large through equation (2.1). We found that early warning signals based on Large s.d. nodes (shown by stars in figure 2) perform better than those based on Large Correlation nodes and that the Large s.d. node set performs approximately as well as the High Input node set. Both the Large s.d. and, to a lesser extent, the Large Correlation node sets perform well even when we consider all node transitions, not just those occurring after a stable range (electronic supplementary material, figure S8). However, the Large s.d. node set is particularly sensitive to the number of samples used to determine node membership; its performance declines substantially on this test when we use only the first 10 samples to determine node membership (electronic supplementary material, figure S7).

Overall, these results support the idea of network-aware choice of sentinel nodes for early warning multi-stage transitions even when we do not have connectivity data at hand.

### 3.4. Transition from the upper state to the lower state

Simulations of equation (2.2) on the power-law and dolphin networks with all nodes beginning in the upper state also show multi-state transitions (see electronic supplementary material, figure S10). With equation (2.2), high-degree nodes receive a large positive contribution from the coupling term, which is the same as with equation (2.1). Therefore, lower-degree nodes or those adjacent to fewer upper-state nodes are most likely to transition from the upper to the lower state when *D* gradually decreases. For this reason, Lower State and High Input, which are two node sets that performed well when we attempted to anticipate transition from the lower to upper states, are not expected to be equally good sentinels when the tipping direction is reversed, that is, when the system begins with nodes at the upper state and transits to the lower state. Therefore, we additionally considered two node sets that are mirror images of Lower State and High Input. One is the set of nodes in the upper state, which we already considered in figure 2. The other is Low Input, which is the *n* nodes with the smallest *R*_*i*_ among the upper-state nodes; they are candidate of nodes that may transit from the upper to the lower state earlier than other nodes as *D* decreases.

**Figure 4.**
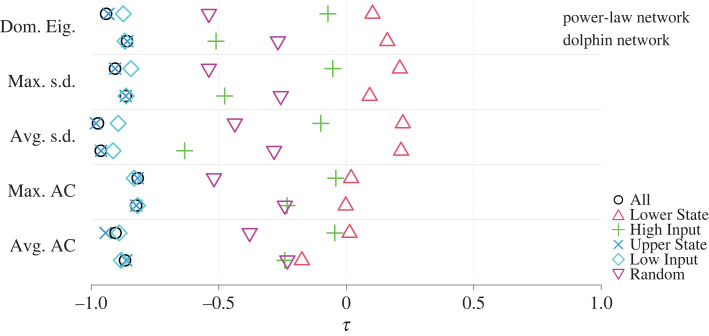
Early warning signals in multi-stage transitions from the upper to lower equilibria. Kendall correlations (*τ*) between each of the five early warning signals and the coupling strength, *D*, for different sets of nodes when the dynamics begin with the nodes in the upper state and *D* gradually decreases are shown. See the caption of figure 2 for the abbreviation of the early warning signals.

We show the Kendall’s *τ* for the power-law and dolphin networks in figure 4. In figure 4, a negative *τ* indicates that the early warning signal became large as *D* decreased towards a transition from the upper to the lower state. Therefore, large negative *τ* values are indicative of critical slowing down as we decrease *D*. We find that the early warning signals calculated from lower-state nodes (Lower State, shown by the triangles and High Input, shown by pluses) are not useful for anticipating transitions. By contrast, those calculated from the All node set (shown by the circles) or those informed by upper-state node dynamics (Upper State, shown by crosses; Low Input, shown by diamonds) are highly negatively correlated with *D*. This result indicates that the nodes in the upper state, not those in the lower state, provide useful early warning signals. Furthermore, the best sentinel nodes are opposite in terms of *R*_*i*_ from when we started with the lower equilibrium and observed transitions of the nodes from the lower to the upper state. A suitable choice of sentinel nodes depends on the tipping direction, even if the dynamical system model is similar or essentially the same.

## 4. Discussion

We showed that both multivariate (i.e. eigenvalue-based) and aggregated univariate (i.e. variance- and autocorrelation-based) early warning signals can provide advance notice of state changes in multi-stage transitions in coupled double-well systems. Furthermore, we showed that constructing early warning signals only based on a subset of nodes, called sentinel nodes, is competitive with, and sometimes more effective than, using all nodes to calculate the early warning signals. Specifically, it is useful to monitor nodes that have not transitioned to the alternative state but are connected to other nodes that have already transitioned to such a state. We showed that the early warning signals calculated based on the thus selected sentinel nodes were effective both when nodes were transitioning from a lower state to an upper state and vice versa. Up to our numerical efforts, the results were robust against parameter variation, network structure and choice of early warning signals.

We have shown that the choice of which nodes to monitor for early warning signals has a marked impact on the effectiveness of the early warning signal. In particular, when we observed transitions from the lower to upper states, a good set of nodes to monitor was those with a large degree or with many connections to other nodes that have already transitioned to the upper state, as quantified by *R*_*i*_. At first glance, this result seems at odds with those by Aparicio *et al.* [14], who used network control theory to propose that lower-degree nodes tended to make better sentinels. In fact, in their model, the dynamics always starts with nodes in the upper state, because it is a model of species abundance and its loss. We showed that lower-degree nodes are good sentinel nodes when the nodes are initially in their upper states and transit to their lower states as a bifurcation parameter gradually changes. Aparicio *et al.* provided two indices for the suitability of their sentinel nodes. Because one of the two indices only depends on the network structure, we calculated the other measure, called *ρ*, for our simulations, given the network. A value of *ρ* closer to zero indicates that their sentinel nodes are more suitable. We found for our power-law network *ρ* = 0.042 when all nodes start in the lower state and *ρ* = 0.038 when all nodes start in the upper state; for the dolphin network, we obtained *ρ* = 0.030 and *ρ* = 0.007, respectively. These results are consistent with our numerical results, in which low-degree nodes provide informative early warning signals when we started with the upper but not the lower state. We emphasize that a good choice of sentinel nodes depends on the initial condition and the tipping direction even if we fix the dynamical system as well as the network structure.

There are many cases in which a network model is thought to represent a complex system showing tipping phenomena but the edges of the network are not directly known [33]. Examples include the co-occurrence of symptoms of neurological conditions [34] and the rates of return on traded financial securities [35]. In such cases, we are typically given only multivariate time-series data and want to derive informative early warning signals for tipping points that possibly constitute a multi-stage transition. A strategy in this situation is to infer the network structure from multivariate time-series data [33,36] and then calculate candidate sentinel nodes from the estimated network using, for example, the node’s ranking in terms of *R*_*i*_. We avoided this approach because network inference from time-series data is subject to error due to e.g. thresholding decisions [33] or uncertainty in model estimation [36]. Instead, we proposed a method to identify sentinel nodes only based on the Pearson correlation between the time-series at pairs of nodes, which provides a proxy to edges (although one should not use the Pearson correlation as an estimate of the network edge in general [37]). Our sentinel nodes determined based on the Pearson correlation provided reasonably strong early warning signals, but their performance did not reach that for the case in which we know the network structure. However, choosing sentinel nodes based on the standard deviation of the node’s state performed in a similar manner to sentinel nodes chosen using information on network structure. Finding better sentinel nodes given multivariate time-series data for which the explicit network structure is unknown warrants future work. We also point out that we currently do not have equivalent methods when the nodes are initially in their upper states and transit to their lower states as the value of a control parameter gradually varies, which is typical in ecological modelling.

Although we have shown that High and Low Input node sets are efficient at anticipating major changes of state in the models we studied, there is much room for further improvements. First, multi-stage transitions imply that there are intermediate stages in which some nodes have tipped and the others have not and that we have seen a history of which nodes have tipped and when. If we use such information, we may be able to improve performances of early warning signals with respect to both the node set selection and the definition of the signal. Second, it may be helpful to use benchmark networks that show multi-stage transitions. If a network is composed of multiple disconnected components of tipping elements, the entire network should show multi-stage transitions because the different disconnected components show a bifurcation at different values of a control parameter in general. Therefore, a network with a strong planted community structure is expected to show multi-stage transitions for various dynamical systems. Degree-heterogeneous random graphs also show multi-stage transitions, which is underpinned by both numerical simulations and a mean field theory [20]. Studying multi-stage transitions and early warning signals on these networks may be useful.

We used cubic polynomials to drive the node’s dynamics (and hence a potential in the form of quartic polynomials) and unipartite networks to test our ideas. These modelling assumptions are reasonable for investigating, for example, climate and vegetation cover transitions [38,39]. By contrast, various ecological systems are better modelled by bipartite networks, in which the two layers of nodes typically represent pollinators (or seed dispersers) and plants [12,40]. In fact, ecological dynamics on bipartite networks also show multi-stage transitions [12]. Despite the seminal work based on network control theory [14], discussed above, further work is desirable for identifying informative sentinel nodes in ecological dynamics on bipartite networks. Other types of dynamics such as reactive and synchronization dynamics on networks should also be investigated. Additionally, although saddle-node bifurcations have been frequently studied, natural systems may also show other types of bifurcations. Early warning signals for transcritical, Hopf and other bifurcations are beyond the scope of this work, but anticipating such transitions is important in several fields, including epidemiology [41] and ecology [12]. Finally, although we have shown that a careful choice of sentinel nodes can dramatically reduce the amount of data needed without sacrificing the quality of early warning signals, we are ignorant of the amount of the data needed from each node in this study. Shortening the length of temporal data required will be an important next step, given that sampling can be expensive and invasive in various applications such as ecology and medicine. Spatial correlations such as Moran’s *I* have been used to provide early warning signals on square lattices [42], and their extensions to the case of complex networks may help reduce the required amount of temporal sampling.

In addition to sampling limitations, the specificity of early warning signals is a known challenge [43–46]. Suppose that an early warning signal tends to increase as a control parameter gradually increases towards a tipping point. It is difficult in general, however, to suggest a particular range of values of the early warning signal that indicates an impending transition. In fact, the Kendall’s *τ*, which is deemed to be a standard performance measure, may be large for several reasons, including when the early warning signal monotonically increases as the control parameter increases regardless of tipping points [44]. This lack of specificity is also present in our results (see figure 1). Developing methods, such as maximum likelihood [44] or algorithmic classification [45] techniques, to improve the specificity of early warning signals is an important area of further research. With all these tasks saved for future work, by combining information about the network structure and dynamics, the present study takes a significant step towards accurately and cost-efficiently anticipating different types of tipping points in complex dynamical systems.

## Data Availability

The datasets generated and analysed during the current study are available in the GitHub repository, https://github.com/ngmaclaren/doublewells, along with all relevant computer code. The data are provided in electronic supplementary material [47].
